# Exploring the role of host specialisation and oxidative stress in interspecific lifespan variation in subtropical tephritid flies

**DOI:** 10.1038/s41598-020-62538-2

**Published:** 2020-03-27

**Authors:** Kévin Malod, C. Ruth Archer, Minette Karsten, Ruben Cruywagen, Alexandra Howard, Susan W. Nicolson, Christopher W. Weldon

**Affiliations:** 1 Department of Zoology and Entomology, University of Pretoria, Private Bag X20, Hatfield, 0028 South Africa; 2 Institute for Evolutionary Ecology and Conservation Genomics, University of Ulm, Ulm, Germany; 3 Department of Conservation Ecology & Entomology, Stellenbosch University, Stellenbosch, South Africa

**Keywords:** Physiology, Entomology

## Abstract

In herbivorous insects, the degree of host specialisation may be one ecological factor that shapes lifespan. Because host specialists can only exploit a limited number of plants, their lifecycle should be synchronised with host phenology to allow reproduction when suitable hosts are available. For species not undergoing diapause or dormancy, one strategy to achieve this could be evolving long lifespans. From a physiological perspective, oxidative stress could explain how lifespan is related to degree of host specialisation. Oxidative stress caused by Reactive Oxygen Species (ROS) might help underpin ageing (the Free Radical Theory of Aging (FRTA)) and mediate differences in lifespan. Here, we investigated how lifespan is shaped by the degree of host specialisation, phylogeny, oxidative damage accumulation and antioxidant protection in eight species of true fruit flies (Diptera: Tephritidae). We found that lifespan was not constrained by species relatedness or oxidative damage (arguing against the FRTA); nevertheless, average lifespan was positively associated with antioxidant protection. There was no lifespan difference between generalist and specialist species, but most of the tephritids studied had long lifespans in comparison with other dipterans. Long lifespan may be a trait under selection in fruit-feeding insects that do not use diapause.

## Introduction

Life-history traits in herbivorous insects are strongly affected by resource availability. For example, insects can improve larval fitness by adapting their timing of oviposition^1^ or synchronising larval hatching to the time when their host plant is most suitable (e.g. budburst)^2^. It is crucial for insects to adapt to the phenology of their preferred host plants, particularly for those that are host specialists and have a narrow window of opportunity to exploit resources. As the probability of encountering a suitable food resource is lower in specialists than in generalists, specialised insects are heavily influenced by temporal host availability. Diapause is one strategy used by insects to cope with environmental instability: they can enter a quiescent state to avoid challenging environmental conditions, where host plants may be unavailable or of low quality, and can synchronise the return to an active state with more favourable conditions^3^. However, for insect species that do not exhibit diapause, or any state of quiescence, but must cope with seasonal variation in resource availability, selection could promote extended lifespan or the widening of host breadth (i.e. becoming generalists). For example, fruit are usually not available all year long, and it has been suggested that fruit-feeding butterflies are under selection for long lifespan^4^. Supporting the idea that long lifespan might be under selection by seasonal availability, a study suggested that populations of adult *Bactrocera opiliae*, which is strictly associated with one host plant, survive approximatively one year without reproducing to reach the next fruiting season^5^. The longer lifespans of insects with a narrower host range may also be connected with the time constraint of searching for a suitable host plant^6^. Differences in lifespan and ageing trajectories between host specialists and generalists have hardly been explored, and the underlying physiological mechanisms for any variation are unknown.

Closely related species can differ tremendously in their lifespan^7^. The mechanistic basis of this interspecific variation in lifespan is still not well understood^8^, but the Free Radical Theory of Ageing (FRTA) has received the most attention as a potential explanation^9–12^. The FRTA states that the accumulation of oxidative damage to cellular components leads to cellular process dysfunction, ageing, and ultimately death^13^. This damage occurs when the production of Reactive Oxygen Species (ROS) exceeds antioxidant defences, which detoxify ROS, or the capacity of the cell to repair oxidative damage. When the balance between ROS production and protection is weighted in favour of ROS generation, the cell enters oxidative stress and oxidative damage occurs. A key prediction of the FRTA is therefore that long-lived individuals or species produce fewer ROS or have better defences or repair mechanisms against ROS. These assumptions are often supported at a sex-specific level, with the longer-lived sex often having lower oxidative damage^14,15^. However, some exceptions have been found in very long-lived species which have neither high antioxidant protection nor low oxidative damage^16–18^. While a number of comparative studies support the FRTA, experimental work has generated equivocal results. For example, studies manipulating the expression of genes coding for antioxidant enzymes have shown no effect on lifespan in transgenic mice^19^. Therefore, the idea that oxidative damage alone underpins ageing and mediates variation in lifespan has largely been abandoned^10,19–21^. Nevertheless, the current consensus is that oxidative damage may be one of several interacting factors involved in causing ageing^22,23^.

Recently, ROS have been incorporated into a trade-off based framework, and researchers have predicted that ROS are a physiological constraint that mediates trade-offs between life-history traits and therefore shapes life-history evolution^11,24–26^. Therefore, given the concept that ROS may be a mediator of lifespan-reproduction trade-offs, any environmental constraint that impacts either trait – lifespan or reproduction – should be associated with the equilibrium between ROS production and antioxidant capacity. One ecological factor that could be associated with this equilibrium is the degree of specialisation, because host plant availability imposes a direct constraint on time of reproduction and lifespan in herbivorous insects.

This study investigates how differences in lifespan are shaped by the degree of host specialisation and then explores the mechanistic basis of these effects, by asking if differences in oxidative damage accumulation in lipids and proteins or variation in antioxidant capacity correlate with lifespan. The role of phylogeny in these traits was also assessed as host specialisation may represent a shared derived character in closely related species^27^. We used eight subtropical true fruit fly species as a model, mostly from the genus *Ceratitis* (Diptera: Tephritidae). This genus comprises at least 100 described species^28^ with a wide variety of host ranges^29^. The range of species we investigated spanned from broad generalists (*Bactrocera dorsalis*, *Ceratitis capitata*, *Ceratitis quilicii*) to extreme specialists (*Ceratitis pedestris*, *Ceratitis podocarpi*, *Ceratitis rubivora*, *Ceratitis scaevolae*) and included a species that could be regarded as a specialised generalist (*Ceratitis cosyra*) based on current host records (i.e. limited number of recorded hosts from different families – see Table S3). We predicted that adult lifespan is constrained by evolutionary history and positively correlated with the degree of specialisation. In other words, specialised species should exhibit a longer lifespan and better ability to maintain the balance between oxidative damage and antioxidant protection to permit survival when hosts are unavailable.

## Results

### Lifespan

**Figure 1:**
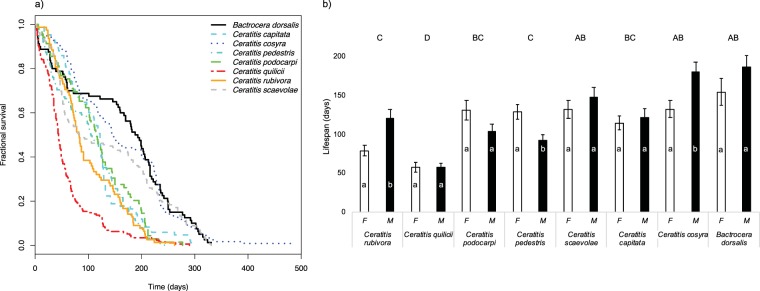
Interspecific comparison of eight species of tephritid flies. (**a**) Survival curves. (**b**) Average lifespan of females and males across species. Sexes within a species with the same lower case letter do not have statistically different survival probabilities (p > 0.05). Species denoted with the same capital letter do not differ significantly from each other (p > 0.05). Error bars indicate the standard error of the mean.

We assayed longevity of individually housed, virgin wild virgin females and males from eight tephritid fruit fly species that fall in one of the following categories of host specialisation: generalist, specialised generalist and specialist. Using parametric survival regression, we found that survival differed between species (χ^2^ = 67.46, df = 7, p < 0.001; Fig. 1a). Amongst the three species with the longest lives, there was a generalist (*Bactrocera dorsalis*), a specialised generalist (*C. cosyra*) and a specialist (*C. scaevolae*) (Fig. 1b). A generalist species, *C. quilicii*, had significantly lower survival than that of all the other species (Fig. 1b). There was no consistent effect of sex on survival (χ^2^ = 1.89, df = 1, p = 0.168), but there was a significant first order interaction between species and sex (χ^2^ = 20.61, df = 7, p = 0.004). This interaction term reflects the observation that, in most species, survival did not differ between sexes; while in *C. cosyra* and *C. rubivora* males lived longest, and in *C. pedestris* females survived longest (Fig. 1b).

### Oxidative damage and total antioxidant capacity

**Figure 2:**
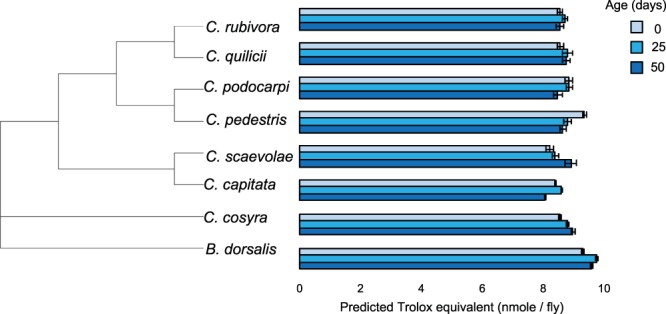
Total antioxidant capacity of eight species of tephritid flies at three different ages and sorted by species relatedness. The values displayed are the Trolox equivalent means predicted by the model used for statistical analyses. Each bar represents 10 individuals, females and males were pooled together (see Fig. S1 for sexes separately). Error bars indicate the standard error of the predicted mean.

For each species, at 0, 25 or 50 days after adult emergence we measured sex specific antioxidant protection (Total Antioxidant Capacity - TAC), and oxidative damage to lipids (i.e. lipid peroxidation) and proteins (i.e. protein carbonyl formation) using colorimetric assays. A generalised linear model showed that there was no difference in TAC between females and males (Table 1). There was a complex interaction between species and age as some species had higher TAC than others depending on the age of the flies (Table 1). Post-hoc tests indicated that at day 0, *B. dorsalis* and *C. pedestris* had higher TAC than *C. cosyra* and *C. capitata*, and *C. pedestris* had significantly higher TAC than *C. rubivora* (Table S1, Fig. 2). However, at days 25 and 50 *B. dorsalis* had higher TAC than all *Ceratitis* species (Table S1, Fig. 2). Moreover, other differences were present at day 50; *C. capitata* had lower TAC than *C. cosyra* and *C. quilicii*. Overall, body mass did not have a significant effect on TAC, but a significant species by body mass interaction indicated that this was not the case in all species (Table 1). Inspection of the coefficients of the slopes indicated that heavier flies had significantly higher TAC in *C. podocarpi* (coefficient = 0.277; p = 0.004), *C. scaevolae* (coefficient = 0.167; p = 0.041) and *C. quilicii* (coefficient = 0.188; p = 0.026), but not in other species.

**Table 1:** Results of linear (lipid peroxidation and protein oxidation) and generalised linear (TAC) analyses on the minimal adequate model.

Effects	Statistic	df	p
**TAC**			
Species	16.64	7	**0.019**
Age	8.53	2	**0.014**
Body Mass	0.08	1	0.775
Species × Age	28.74	14	**0.011**
Species × Body Mass	17.77	7	**0.013**
**Lipid peroxidation**			
Species	5.71	7	**<0.001**
Sex	6.46	1	**0.011**
**Protein oxidation**			
Species	2.82	7	**0.008**
Protein content	21.67	1	**<0.001**

**Figure 3:**
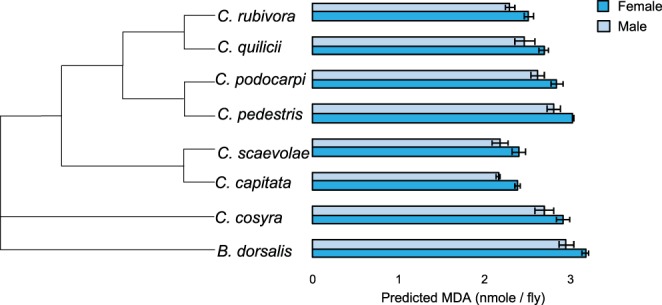
Lipid peroxidation in females and males of eight species of tephritid flies sorted by species relatedness. The values displayed are the MDA means predicted by the model used for statistical analyses. Each bar represents 15 individuals, age categories were pooled together (see Fig. S1 for ages separately). Error bars indicate the standard error of the predicted mean.

Using a linear model, we found that species and sex significantly affected lipid peroxidation (Table 1). Females experienced more oxidative damage to lipids than males (Table 1, Fig. 3). Post-hoc multiple comparisons showed that *Bactrocera dorsalis* and *C. pedestris* had significantly more oxidative damage to lipids than *C. capitata* (p < 0.001; p = 0.007) and *C. scaevolae*, (p < 0.001; p = 0.008), and *B. dorsalis* also experienced more damage than *C. rubivora* (p = 0.005) (Fig. 3). Age and lipid reserves did not affect lipid peroxidation.

**Figure 4:**
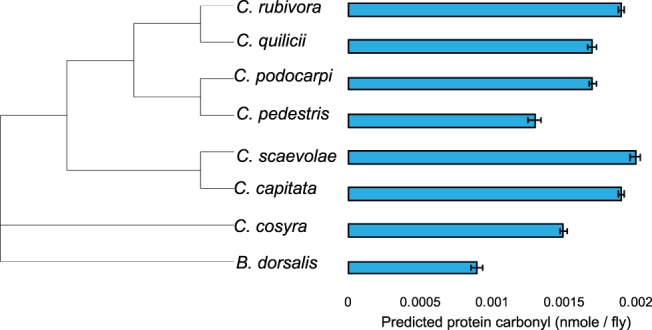
Protein oxidation of eight species of tephritid flies sorted by species relatedness. The values displayed are the protein carbonyl group formation means predicted by the model used for statistical analyses. Each bar represents 30 individuals, age categories and sexes were pooled together (see Fig. S1 for ages and sexes separately). Error bars indicate the standard error of the predicted mean.

The outputs of a linear model showed that oxidative damage to protein differed between species (Table 1). Post-hoc tests revealed that *B. dorsalis* had the lowest protein oxidation (Fig. 4), and *C. pedestris* had lower damage than most other *Ceratitis* species (*C. capitata*, *C. podocarpi*, *C. rubivora* and *C. scaevolae*). In addition, we found that protein oxidation was lower in flies that stored more soluble protein (coefficient = −1.33 × 10^−6^, p < 0.001) (Table 1). Sex and age did not affect oxidative damage to protein.

### Relationship between oxidative damage, antioxidant protection, phylogeny and lifespan

**Figure 5:**
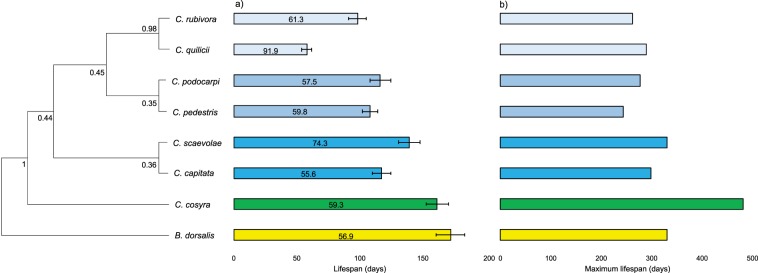
Average (**a**) and maximum lifespan (**b**) of eight species of tephritid flies sorted by their relatedness. The coefficient of variation for lifespan is indicated in the bars representing average lifespan (**a**). Error bars indicate the standard error of the mean. The numbers at each node of the maximum likelihood phylogenetic tree represent the value of the bootstrap to assess the robustness of each branch. The maximum lifespan observed was achieved by a male *C. cosyra* that reached 481 days.

We used the mitochondrial cytochrome c oxidase I (COI) gene to establish the relatedness between the eight species used and construct a maximum likelihood phylogenetic tree. Then, we checked how the different variables for lifespan were affected by the physiological parameters measured, the number of host plants and body weight. Subsequently, we added species relatedness to the model to assess the effect of phylogeny. Using ordinary least-squares regression, we found that average lifespan slightly but significantly increased as TAC increased (Table 2), but the use of phylogenetic least-squares regression showed that this was not related to species relatedness (Fig. 5a). In addition, host plant range had no effect on average lifespan. None of the parameters recorded in this study had an effect on maximum lifespan, with the null model best describing maximum lifespan (Table 2), and maximum lifespan was not associated with phylogeny (Fig. 5b). We found that the variation in lifespan was lower in species with higher TAC, in contrast, and heavier species had greater variation in lifespan (Table 2), and there was no effect of the number of host plants. Similar to average lifespan and maximum lifespan, we did not detect any effect of species relatedness on the coefficient of variation for lifespan (Fig. 5a). Finally, none of the factors we recorded had an effect on survival to 50 days, and survival was not constrained by phylogeny (Table 2).

**Table 2:** Coefficients from the minimal adequate ordinary least-squares regression (OLS) and phylogenetic least-squares regression (PGLS) models for average lifespan, maximum lifespan, coefficient of variation for lifespan, and survival to fifty days of eight tephritid fly species. The minimal adequate model was determined from the OLS based on the best AIC and using step-wise deletion of the terms. Phylogenetic correlation for each trait is given by the value of λ, with zero indicating no phylogenetic signal and one indicating a strong signal.

Effects	OLS				PGLS				
	Estimate	t	p	AIC	Estimate	t	p	AIC	λ
**Lifespan**									
(Intercept)	99.08	3.73	**0.013**	77.98	99.05	3.65	**0.014**	76.17	0
Protein damage	−0.07	−1.32	0.241		−0.07	−1.3	0.248		
TAC	0.01	2.71	**0.042**		0.01	2.68	**0.043**		
**Maximum lifespan**									
(Intercept)	314.12	11.99	**<0.001**	94.51	314.44	11.85	**<0.001**	92.73	0
**Coefficient of variation**									
(Intercept)	77.68	9.58	**<0.001**	59.09	52.41	5.01	**0.007**	57.16	0
Host	−0.037	−1.11	0.328		−0.037	−1.11	0.330		
TAC	−0.005	−3.99	**0.016**		−0.005	−3.99	**0.016**		
Body mass	4.99	2.89	**0.044**		4.99	2.89	**0.044**		
**Survival to 50 days**									
(Intercept)	2.71	2.70	0.114	−7.82	2.71	2.69	0.115	−9.72	0
Host	0.004	2.14	0.166		0.004	2.13	0.167		
Protein damage	−0.002	−1.64	0.243		−0.002	−1.63	0.245		
Lipid damage	0.017	1.63	0.244		0.017	1.62	0.247		
TAC	0.0001	1.75	0.222		0.0001	1.75	0.223		
Body mass	−0.184	−1.93	0.193		−0.184	−1.93	0.193

## Discussion

In this study, we investigated variation in lifespan between eight fruit fly species as a function of their degree of host specialisation and relatedness. We also measured oxidative damage to lipids and proteins, as well as antioxidant protection to assess whether lifespan differences are associated with oxidative stress. We have three key findings. The variation in lifespan between these closely related fruit fly species was not related to the degree of specialisation. Oxidative stress may play a role in mediating this variation as species with better antioxidant protection tended to live longer. However, these long-lived species did not have lower oxidative damage, showing that if oxidative damage influences lifespan the relationship is complex.

In many fly species, lifespan is usually counted in days or weeks. In contrast, the first observation in this study is that most of the tephritid species investigated had exceptionally long lifespans. With the exception of *C. quilicii*, all the species lived on average more than three months, and some individuals survived for more than a year. This is significantly greater than other dipterans that are smaller (e.g. *Drosophila melanogaster*^30^, *Anopheles gambiae*^31^) or much larger (e.g. *Glossina pallipides*^32^, *Calliphora stygia*^33^) but live for two months or less. Even in comparison with flies of similar size (e.g. *Musca domestica*^34^), the species investigated here lived two to five-fold longer. Among tephritid species there have been other reports of extremely long lives, with lifespans of up to 431 and 357 days for *Anastrepha alveata* and *Ceratitis rosa* respectively^35,36^. Apart from host range, little is known about the biology of many species used in this study. Among species for which observations do exist in the literature, notable differences were observed in *C. quilicii* and *B. dorsalis*. Wild *C. quilicii* from South Africa in this study had a shorter average lifespan than laboratory reared flies from Kenya at a similar temperature^37^. This may be due to intra-specific variation between populations, as average lifespan of a species can differ significantly depending on its geographic origin^38^. It has been reported that in some ectotherm species from various taxa (including insect species), latitude can explain intra-specific variation in lifespan between populations from different locations^39^. As Kenya is closer to the equator than South Africa, it may be that lifespan differences observed between the studies on *C. quilicii* are related to differences in the latitude of the locations. In addition, the average lifespan of *B. dorsalis* was much longer than reported from other studies of either laboratory-adapted^40^ or wild-collected^41^ individuals. This difference may relate to rearing temperature in these other studies, 27 and 28 °C respectively, because in other fruit fly species adult lifespan is affected by temperature and the temperature-dependent trade-off between lifespan and reproduction^37,42^. Moreover, there was no phylogenetic signal for lifespan, indicating that it was not determined by species relatedness. This contrasts with higher taxonomic levels where the phylogenetic signal for lifespan is present and strong^43^. The strength of the phylogenetic signal could decrease when moving towards lower taxonomic levels and the results of the present study may show that lifespan is not affected by species relatedness when comparing within a genus.

Despite exceptionally long lifespans being achieved in almost all species, three were particularly long-lived. Counter to expectation, these three species covered the full spectrum of the degree of specialisation, with a generalist (*B. dorsalis*), a specialised generalist (*C. cosyra*) and an extreme specialist (*C. scaevolae*) represented. If we consider only the *Ceratitis* species, the generalist *C. quilicii* was the shortest-lived species in this study, and the two longest lived were specialists. However, the generalist species *C. capitata* lived as long as other specialists. Moreover, with an average lifespan of 140 days, the generalist species *C. rosa*^36^ would sit between *C. cosyra* and *C. scaevolae*, giving more support to the fact that lifespan in this genus may not be determined by host specialisation. Confirming these observations, when regressions corrected by phylogeny were used, the number of hosts did not affect average lifespan, maximum lifespan or the coefficient of variation. Nevertheless, we have previously suggested that the trade-off between lifespan and reproduction might not be as strong in specialist species (e.g. *C. cosyra*) as it is in generalist species^44^. As virgin flies were used and females had no substrate to lay eggs, the difference in lifespan that was expected between specialists and generalists may have been masked due to effects on oocyte maturation. There is evidence in the literature that *C. capitata* exhibit continuous maturation of oocytes, whereas oocyte maturation in *C. cosyra* may be discontinued when no suitable host plants are available^45^. Further investigations should take into account mating status and type of oogenesis (continuous versus discontinuous development) as host plant breadth might play a role in oocyte maturation^46^, which then increases oxidative stress^47^. Alternatively, the absence of differences due to degree of specialisation might have been biased by the definition of specialist or generalist species itself, as here it relates to the number of host plants exploited by a species. For example, in drosophilids it was suggested that instead of selecting host plants, insects may rather select the microbiota inhabiting the host^48^, which would decrease the number of species categorised as specialists. The phenology of the plant species rather than the number of available hosts might explain some interspecific variance in lifespan. However, data on fruit phenology are not available for some indigenous South African plant species and therefore, it is not possible to test this hypothesis.

Regardless of the degree of specialisation, and aligning with observations in non-diapausing tropical fruit feeding butterflies^4^, our results suggest that sub-tropical tephritid flies not experiencing diapause that use fruit as a larval diet are under selection for long adult lifespan to cope with fruit availability. Therefore, latitude, which influences temperature but also whether a species is diapausing or not and the type of food resources used, may be of greater influence on adult lifespan than the degree of specialisation. Nevertheless, as reproduction usually shortens lifespan in fruit flies, there is a need to investigate lifespan differences in mated specialist and generalist species. The long lifespans of the fruit fly species investigated here may be a feature of individuals waiting for host availability and reproduction, thereafter life expectancy may drop in a different manner between species once suitable fruits are available and individuals mated^49^. Observations in *B. opiliae* suggest that this specialist species can achieve extremely long lifespan, but that mortality increases just after the peak of host availability when most females have reproduced and laid eggs^5^. In addition, the fruit phenology (see Table S5) of the host plants associated with each fruit fly species used in this study shows that, in South Africa, they experience long periods of fruit unavailability, whether due to hosts fruiting only for a short period of time, or because fruiting is sporadic^50^. Therefore, at least a part of the population must achieve long lifespans to reach the next fruiting period, which aligns with our results. For instance, *C. quilicii* lives on average less than two months but the maximum lifespan of some individuals can be five-fold longer.

Under the assumptions of the FRTA, the longest-lived species should be the best at maintaining the equilibrium between ROS and antioxidant protection. Foremost, this should mean that species that live longest have the lowest oxidative damage. Our results provided mixed support for the theory. The three longest-lived species were not necessarily the species suffering the least lipid peroxidation or protein oxidation, with *C. scaevolae* having one of the lowest levels of lipid peroxidation, *B. dorsalis* experiencing the most damage to lipids, and *C. cosyra* being intermediate between the other two species. Conversely, the lowest protein oxidation was found in *B. dorsalis*, and *C. scaevolae* was one of the species most affected by oxidative damage to protein. Across all species, oxidative damage to lipids and proteins was generally very similar. The current results are in contrast with observations in colubrid snakes, where ROS production in the mitochondria was measured, and it was found that ROS production was reduced in the long-lived species^7^. However, the snake species used by Robert *et al*.^7^ were from the same family but different subfamilies and genera to avoid phylogenetic grouping, which may explain higher phenotypic variation of the trait in comparison with the present study.

Here, we found that the species with the best overall antioxidant protection, *B. dorsalis*, was one of the longest-lived. However, long-lived species from the genus *Ceratitis*, such as *C. cosyra* and *C. scaevolae*, did not have outstanding TAC in comparison with the other species with shorter lifespans. This aligns with findings in vertebrates where exceptional lifespan is not associated with exceptional antioxidant protection^16–18^. Nevertheless, average lifespan significantly increased with TAC, and this was not due to the phylogenetic relationship between species. In addition, the variation in lifespan decreased significantly in species with higher TAC level. The positive association between lifespan and TAC partially mirrors observations in birds from different families, where higher TAC was associated with higher maximum lifespan^51^. In Vágási *et al*.^51^, species were from different families, whereas in the present study, except the external genus *Bactrocera* used to root the phylogenetic tree, species belonged to the same genus. Therefore, as shown by the results, the variation in TAC between species was small and the maximum lifespans observed were in most cases very close.

Finally, if ageing is caused by accumulation of oxidative damage, either oxidative damage or antioxidant protection, or both at the same time, should change with age. This idea was not supported by the present data. Oxidative damage to either protein or lipid did not accumulate at older age; instead, oxidative damage and antioxidant protection remained constant across ages. These observations are somewhat similar to results reported in zebra finches, *Taeniopygia guttata*. In those birds, no change in ROS production across ages was observed, but unlike in the present study, TAC was found to be higher at intermediate ages^52^. Nevertheless, as suggested by the lifespan data, apart from in *C. quilicii*, 50 days is not very old for the seven other fruit fly species. Therefore, if no change in oxidative damage and TAC has been observed across the three age categories, it may be that accumulation of oxidative damage arises beyond 50 days of age, and antioxidant protection would increase accordingly to prevent or limit damage. Further investigations are needed to track oxidative damage and TAC at older ages in these long-lived species.

In conclusion, this study shows that at low taxonomic levels lifespan is not correlated with the degree of specialisation, nor with species relatedness. Instead, the results suggest that long lifespan may be an evolutionary strategy under selection in non-diapausing, fruit-feeding insects. Regarding the equilibrium between ROS and antioxidant protection, this study provided mixed support for the FRTA. In contrast with one of the FRTA assumptions, oxidative damage did not accumulate with age, but instead remained constant. In addition, short-lived species did not experience more oxidative damage. However, across species, average lifespan increased with TAC, regardless of the degree of specialisation or species relatedness.

## Methods

### Fruit sampling and fruit fly species

In this study, we used eight species from the family Tephritidae that are either native or invasive in South Africa; seven from the genus *Ceratitis* and one from the genus *Bactrocera* to root the phylogenetic analysis. There are no records from the literature suggesting that any of these species enter diapause at larval or pupal stages. In order to obtain tephritid fruit flies, different species of fruit known for their host status were collected from various locations (Table S2) in South Africa. While some of these species originate in the tropics, all populations were collected in a subtropical region and therefore are referred to as such in this study. Fruits were brought back to the Hatfield campus of the University of Pretoria, South Africa, and stored in a climate room at ~23 °C with a 14:10 light:dark photoperiod. They were placed in a 38.5 L plastic box that was covered with white voile curtain fabric and filled with a 10 mm-deep layer of sand. In order to retrieve pupae, sand for each species of fruit was sifted daily. Collected pupae were placed individually in a 125 mL plastic container covered with insect screen. In each container we placed the lids of two 2 mL microcentrifuge tubes filled with sucrose or hydrolysed yeast (Yeast Extract Powder, Biolab, Merck, Germany). Filtered water was provided through the insect screen in a 200 µL pipette tip loosely capped at the wide end with putty-like pressure-sensitive adhesive to minimise evaporation. Emerging adults were sexed based on the presence or absence of an ovipositor (females) and identified using morphological keys^28,53^.

### Lifespan

The date of adult emergence was recorded. Thereafter, mortality was checked daily and water and food replaced when close to being depleted. We recorded adult lifespan of females and males for each species (see Table S3 for group size – sample size was constrained by the number of flies emerging front fruit). The longevity assay took place in the same plastic container where pupae were initially placed so all individuals were virgin and maintained individually. Keeping flies individually allowed us to control for variation in lifespan that might be driven by mating frequency, the cost of male harassment or male-male aggression both across the sexes and between species.

### Oxidative damage and total antioxidant capacity

From the flies that were set up for the longevity assay, we collected for each species 5 females and 5 males at three different ages (0, 25 and 50 days). Flies were chilled at −20 °C for several minutes before being transferred to individual 2 mL microcentrifuge tubes, which were then stored in a −80 °C freezer. This led to a total of 240 flies being stored for assays for oxidative damage and antioxidant capacity.

To measure oxidative damage to proteins and lipids and total antioxidant capacity (TAC) we used commercial kits (Sigma Aldrich, Saint-Louis, MO, USA) and adapted the supplier recommendations. To measure body composition (soluble proteins, lipids) we used the methods described by Foray, *et al*.^54^ that have been adapted for tephritids^55^. By doing so, we were able to measure all biochemical parameters in a single fly. Soluble proteins and lipid reserves were measured to determine if oxidative damage to proteins and lipids changes as a function of body composition. All assays were colorimetric, where determination of the component of interest was performed by reading absorbance of samples and comparing it to a standard curve with a microplate reader (Eon Microplate Spectrophotometer, Biotek Instruments, Winooski, VT, USA). Ten microplates were necessary to determine each biochemical parameter for the 240 flies. Flies from each combination of species, sex and age were haphazardly assigned to each microplate to avoid potential variation between plates.

Flies were removed from the −80 °C freezer, weighed to determine wet mass and individually placed in a 2 mL screwcap tube with a 3 Ø zirconium bead and 200 µL of 5 mM phosphate-buffered saline (PBS) buffer (1.35 mM potassium chloride, 68.5 mM sodium chloride, pH 7.4) (P4417, Sigma Aldrich, Saint-Louis, MO, USA). Flies were then homogenised using a benchtop homogeniser for one minute. After homogenisation, samples were centrifuged at 200 RCF for 15 min at room temperature (~20 °C). After centrifugation, 100 µL of the homogenised solution was transferred to a new 2 mL microcentrifuge tube for later determination of oxidative damage to protein. To the remaining homogenate, 500 µL of PBS buffer was added for later determination of TAC, lipid peroxidation, and lipid and protein content.

The TAC assay kit (Sigma Aldrich, MAK187) determines the total, enzymatic and non-enzymatic antioxidant capacity. To determine TAC, we placed 10 µL of fly homogenate directly into a well of a 96-well microplate and added 90 µL of PBS buffer to have a ten times dilution. Then 100 µL of Cu^2+^ working solution was added to each sample. To protect from evaporation and light, the microplate was covered with a transparent plastic film and a piece of aluminium foil, and then incubated at room temperature for 90 min. After incubation, the film and foil were removed. Sample absorbance was read at 570 nm and compared to 6-hydroxy-2,5,7,8-tetramethylchroman-2-carboxylic acid (trolox; a water-soluble vitamin E analog) standards (0, 20, 40, 60, 80 and 100 nmole/µL). TAC in a fly was expressed in nmole of trolox equivalent per fly.

For the lipid determination assay, 180 µL of fly homogenate was transferred to a new 2 mL microtube. Then, 20 µL of 20% Na_2_SO_4_ and 1.5 mL of chloroform:methanol (1:2 v/v) were added to the samples and vigorously combined using a vortex mixer. After being vortexed, samples were centrifuged at room temperature for 15 min at 200 RCF. For lipid determination we transferred in duplicate 100 µL of supernatant to a new microtube. Samples were then evaporated under a fume hood for 24 h. After evaporation, 10 µL of 98% sulphuric acid was added and samples incubated at 90 °C for 2 min. After incubation, samples were cooled on ice for 10 min. Then, 210 µL of vanillin reagent (1.2 g/L in 68% orthophosphoric acid) was added to the samples. After addition of the reagent, samples were shaken for 15 min at room temperature and absorbance read at 525 nm. Absorbance of samples was compared with glycerol trioleate standards (0, 0.1, 0.2, 0.5 and 1 mg/mL).

Oxidative damage to lipid was assessed by determination of lipid peroxidation through measurement of malondialdehyde (MDA) formation (Sigma Aldrich, MAK085 kit). From the fly homogenate, 200 µL was pipetted and placed in a new 2 mL microtube. Then 3 µL of butylated hydroxytoluene (BHT) and 150 µL of MDA lysis buffer solution as well as 153 µL of 2 N perchloric acid were added to the 200 µL solution. Samples were then centrifuged at 13 000 RCF for 10 min. A volume of 200 µL from the supernatant was then transferred to a new 2 mL microtube and 600 µL of thiobarbituric acid (TBA) solution was added. Samples were then incubated for 60 min at 90 °C and cooled on ice for 10 min. Sample absorbance was read at 532 nm and compared against MDA standards (0, 0.5, 1, 2, 4 and 6 nmole). Oxidative damage to lipids should be expressed in nmole of MDA per mg of lipids. However, because lipid content was too low to be detected in some samples, we expressed the oxidative damage to lipids in nmole of MDA per fly.

Protein content was determined using a bicinchoninic acid protein kit (Sigma Aldrich, B9643). The 600 µL homogenized solution was centrifuged for 5 min at 3000 RCF and 20 µL of the supernatant transferred to a new 1.5 mL microtube. To have a two times dilution, 20 µL of PBS buffer was added to 20 µL of the homogenized solution. Then, 200 µL of BCA working reagent (bicinchoninic acid solution with copper (II) sulfate pentahydrate, 4% solution) was added to the 40 µL of diluted samples. Samples were then shaken for 30 s and incubated for 30 min at 37 °C. After incubation, sample absorbance was read at 562 nm and compared with the absorbance of bovine serum albumin (BSA) standards (0, 25, 125, 250, 500, 750 and 1000 µg/mL).

Protein oxidation was measured by determining protein carbonyl group content with a kit (Sigma Aldrich, MAK094). To the 100 µL previously set aside, 10 µL of 10% streptozocin was added and samples incubated for 15 min at room temperature. They were then centrifuged at 13 000 RCF for 5 min and the supernatant from each tube was transferred to a new 1.5 mL microtube. To the supernatant, 100 µL of 2, 4-dinitrophenylhydrazine solution was added and samples incubated for 10 min at room temperature. After incubation, 30 µL of 100% trichloroacetic acid solution was added and samples were then placed on ice and incubated for 5 min. Following incubation, samples were centrifuged for 5 min at 13 000 RCF and the supernatant was then discarded. To the remaining pellet, 500 µL of ice cold 100% acetone was added and samples were placed for 30 s in an ultrasonic bath (40 Hz) (DU-32, Argo Lab, Italy) at room temperature. Then, samples were incubated at −20 °C for 5 min. After incubation, samples were centrifuged at 13 000 RCF for 2 min and the acetone was removed. All steps from adding 500 µL of acetone to removing it were repeated. After discarding the second acetone wash, 210 µL of 6 M guanidine solution was added to the pellet and samples placed in the ultrasonic bath for 30 s. Absorbance of samples was read at 375 nm and compared to the absorbance of ultra-pure (double distilled and deionised) water. Oxidative damage to protein was expressed in nmole of protein carbonyl per fly.

### Phylogeny

To assess the phylogenetic relatedness between sampled species, we used the mitochondrial cytochrome c oxidase I (COI) gene. Sequences for *C. scaevolae* were provided by the Royal Museum for Central Africa, Belgium, and the sequences for the other species were downloaded from GenBank or BOLD (Table S4). Sequences were then aligned using MAFFT^56^. If needed, extremities of the sequences were cut in BioEdit 7.0.5^57^ to align sequence length to the shortest one so that all sequences had the same number of nucleotides. The best model of sequence evolution corresponding to our data was determined in MEGA X^58^ based on Akaike’s Information Criterion (AIC) scores. Thereafter, a maximum likelihood phylogenetic tree was drawn in the same software, with the statistical support for branches determined by a thousand bootstraps.

### Statistical analyses

All data analyses were performed using the R v. 3.5.0 statistical environment (R Core Team, 2018, Vienna, Austria).

To determine the effect of species and sex on survival, a survival analysis was performed using the function “survreg” from the “survival” package^59^. The “step” function in R was used to determine, based on the lowest AIC, the best model by removing the least influential parameters. If a significant main effect was found, *post hoc* pairwise comparisons tests of the estimated marginal means were performed using the function “emmeans” from the R package of the same name^60^.

A linear model with the main effects of species, sex and age, and soluble protein content as a covariate was used to explain the variation in protein oxidation. Interactions between all terms were included in the model, except for the covariate where only the interaction with species was added to consider the potential effect of size differences among species. If an interaction was detected, Tukey *post-hoc* multiple comparisons tests were used to identify differences between groups. The same approach was used to explain the variation in lipid oxidation, except that soluble protein content was replaced by lipid content as the covariate. To use linear models, data for protein and lipid oxidation were transformed using 1/x and log(x + 1) respectively. For graphical representation we plotted the means predicted by the model.

Data transformation did not help to normalise the data distribution for total antioxidant capacity. Due to this, we used a generalised linear model with Gamma family and log-link. Because zero values prevent use of the Gamma family, if the detected TAC for an individual was zero, this value was replaced with the smallest value of the dataset and divided by ten. The main effects added to the model were species, sex and age, and adult body mass was added as a covariate. Interactions between all terms were added to the model, except for the covariate where only the interaction with species was added to take into account size differences among species. In order to compare between groups after using the generalised linear model, if a significant interaction was detected, *post hoc* multiple comparisons tests of the means with Tukey contrasts were performed. This was done using the function “glht” in the R package “multcomp”^61^. For graphical representation the means predicted by the model were plotted.

To determine how lifespan was influenced by species relatedness, number of hosts, physiological parameters (TAC, protein and lipid oxidation) and body weight, we employed the approach used by Weldon *et al*.^62^. The number of hosts (Table S2) refers to the number of confirmed host plants (i.e. flies were reared from the fruit). For body mass of each species we used the wet weight measured on the flies from the biochemical assays. We excluded 0 day old flies, as they are not sexually mature and therefore significantly lighter than their older counterparts are^63^. Separate ordinary least-squares regression (OLS) analyses were used to relate the number of hosts, mean body mass (age 25 and 50 pooled together), mean lipid and protein oxidation, as well as mean TAC for each species, regardless of the age, to maximum lifespan, lifespan coefficient of variation or average lifespan. The coefficient of variation was used to indicate variation in lifespan within species on a common scale. A low coefficient of variation would indicate that lifespan is a well conserved trait within a given species. We used the “step” function in R to perform step-wise deletion and determine for each response variable the minimal adequate model based on the best AIC. Then to take species relatedness into account, we used phylogenetic least-squares regression (PGLS) using the R package “caper”^64^ and the function “pgls”. The same minimal adequate model previously determined for the OLS was used for the PGLS. A maximum likelihood approach was used for the PGLS, and the strength of the phylogenetic signal was determined based on the value of λ ranging from zero (no phylogenetic signal) to one (strong phylogenetic signal). The same approach was repeated to relate the number of hosts, mean body weight, mean lipid and protein oxidation, as well as mean TAC for each species at 50 days old to the fraction of flies (i.e. number of flies still alive at 50 days / total number of flies for a given species) that survived 50 days or more.

## Supplementary information

Supplementary information.

## Data Availability

Data are available from the University of Pretoria repository: 10.25403/UPresearchdata.11941314.v1
