# Supplementary material for: Exploring the role of host specialisation and oxidative stress in interspecific lifespan variation in subtropical tephritid flies
Source: Sci Rep. 2020 Mar 27;10:5601. doi: 10.1038/s41598-020-62538-2 (PMC7101423; doi:10.1038/s41598-020-62538-2)
Supplement: Supplementary file 1 — Supplementary information. [file 41598_2020_62538_MOESM1_ESM.pdf]

SUPPLEMENTARY INFORMATION

Exploring the role of host specialisation and oxidative stress in interspecific lifespan variation in subtropical tephritid flies

Kevin Malod, C. Ruth Archer, Minette Karsten, Ruben Cruywagen, Alexandra Howard, Susan W. Nicolson, Christopher W. Weldon.

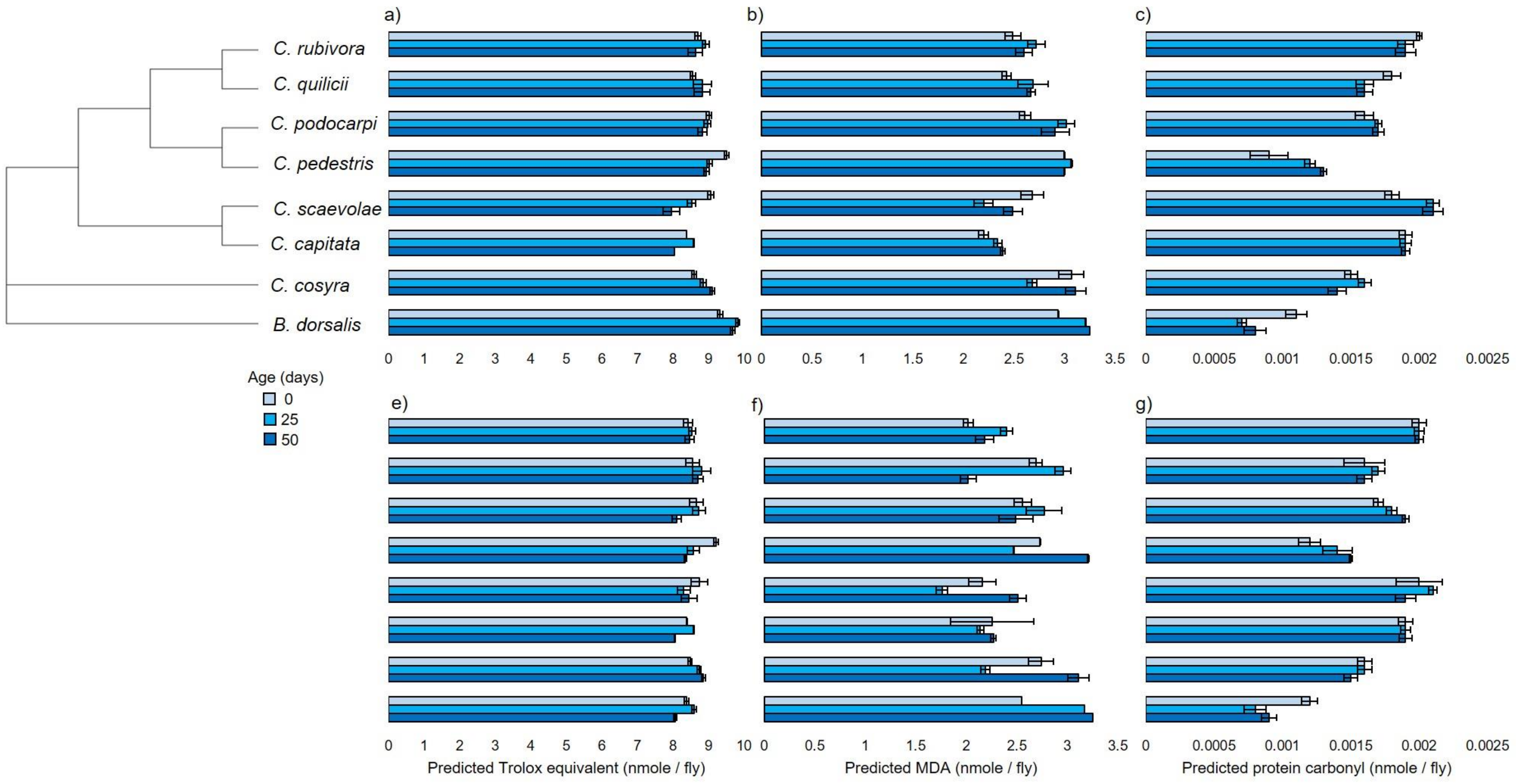

Figure S1. Oxidative damage and antioxidant protection of eight species of tephritid flies at three different ages and sorted by species relatedness. Total antioxidant capacity in females (a) and males (e). Lipid peroxidation in females (b) and males (f). Protein oxidation in females (c) and males (g). The values displayed are the means predicted by the model used for statistical analyses. Error bars indicate the standard error of the predicted mean.

Table S1. Post-hoc comparisons for total antioxidant capacity across species at specific ages (days).

Species 1	Species 2	Age	Estimate	z	p
Cosyra	*Capitata*	0	0,156	0,76	1
		25	0,228	1,13	1
		50	0,928	4,49	**< 0.01**
Dorsalis	*Capitata*	0	0,925	4,47	**< 0.01**
		25	1,168	5,65	**< 0.001**
		50	1,566	7,58	**< 0.001**
Pedestris	*Capitata*	0	0,973	4,71	**< 0.001**
		25	0,290	1,40	0,999
		50	0,638	3,08	0,241
Podocarpi	*Capitata*	0	0,442	2,14	0,898
		25	0,341	1,65	0,994
		50	0,524	2,53	0,648
Quilicii	*Capitata*	0	0,336	1,63	0,995
		25	0,194	0,94	1
		50	0,780	3,77	**0,03**
Rubivora	*Capitata*	0	0,193	0,93	1
		25	0,172	0,83	1
		50	0,565	2,74	0,487
Scaevolae	*Capitata*	0	0,699	3,38	0,11
		25	-0,196	-0,97	1
		50	0,227	1,10	1
Dorsalis	*Cosyra*	0	0,768	3,72	**0,036**
		25	0,940	4,65	**<0.001**
		50	0,637	3,08	0,244
Pedestris	*Cosyra*	0	0,816	3,95	**0,016**

		25	0,061	0,30	1
		50	-0,290	-1,40	0,999
		0	0,285	1,38	0,999
Podocarpi	*Cosyra*	25	0,112	0,55	1
		50	-0,405	-1,96	0,957
		0	0,180	0,87	1
Quilicii	*Cosyra*	25	-0,030	-0,17	1
		50	-0,148	-0,72	1
		0	0,036	0,17	1
Rubivora	*Cosyra*	25	-0,055	-0,28	1
		50	-0,363	-1,75	0,98
		0	0,543	2,63	0,574
Scaevolae	*Cosyra*	25	-0,425	-2,15	0,891
		50	-0,701	-3,39	0,107
		0	0,048	0,23	1
Pedestris	*Dorsalis*	25	-0,878	-4,25	< **0.01**
		50	-0,927	-4,48	< **0.01**
		0	-0,482	-2,33	0,794
Podocarpi	*Dorsalis*	25	-0,828	-4,01	**0,013**
		50	-1,042	-5,04	< **0.001**
		0	-0,587	-2,84	0,406
Quilicii	*Dorsalis*	25	-0,974	-4,71	< **0.001**
		50	-0,780	-3,80	**0,028**
		0	-0,732	-3,54	0,07
Rubivora	*Dorsalis*	25	-0,996	-4,82	< **0.001**
		50	-1,000	-4,84	< **0.001**
Scaevolae	*Dorsalis*	0	-0,225	-1,09	1

		25	-1,365	-6,76	**< 0.001**
		50	-1,339	-6,47	**< 0.001**
		0	-0,531	-2,57	0,623
Podocarpi	*Pedestris*	25	0,050	0,24	1
		50	-0,114	-0,55	1
		0	-0,636	-3,08	0,247
Quilicii	*Pedestris*	25	-0,095	-0,46	1
		50	0,142	0,69	1
		0	-0,780	-3,77	**0,03**
Rubivora	*Pedestris*	25	-0,117	-0,57	1
		50	-0,073	-0,35	1
		0	-0,273	-1,32	0,999
Scaevolae	*Pedestris*	25	-0,486	-2,41	0,743
		50	-0,411	-1,98	0,95
		0	-0,105	-0,51	1
Quilicii	*Podocarpi*	25	-0,145	-0,71	1
		50	0,256	1,24	0,999
		0	-0,249	-1,21	1
Rubivora	*Podocarpi*	25	-0,168	-0,81	1
		50	0,041	0,20	1
		0	0,257	1,25	0,999
Scaevolae	*Podocarpi*	25	-0,537	-2,66	0,546
		50	-0,296	-1,43	0,999
		0	-0,144	-0,70	1
Rubivora	*Quilicii*	25	-0,022	-0,11	1
		50	-0,215	-1,04	1
Scaevolae	*Quilicii*	0	0,363	1,75	0,988

		25	-0,391	-1,94	0,962
		50	-0,553	-2,67	0,535
		0	0,507	2,45	0,712
Scaevolae	*Rubivora*	25	-0,369	-1,83	0,98
		50	-0,338	-1,64	0,995

Table S2. Locations of host fruits from which the experimental flies originated.

	Fruit		Location	
**Fly species**	**Species**	**Family**	**Locality in South Africa**	**Coordinates**
Bactrocera dorsalis	*Mangifera indica*	Anacardiaceae	Low's Creek, Mpumalanga	25˚41'09.2"S 31˚11'05.3"E
Ceratitis capitata	*Psidium guajava*	Myrtaceae	Stellenbosch, Western Cape	33˚52'57.7"S 18˚44'28.7"E
Ceratitis quilicii	*Harpephyllum caffrum*	Anacardiaceae	Pretoria, Gauteng	25˚46'44"S 28˚13'24"E
	Syzigium jambos	Myrtaceae	Pretoria, Gauteng	25˚45'09.2"S 28˚13'46.6"E
Ceratitis cosyra	*Sclerocarya birrea*	Anacardiaceae	Bultfontein, Gauteng	25˚28'54"S 28˚13'19"E
Ceratitis rubivora	*Rubus fruticosus*	Rubiaceae	Hilton, KwaZulu-Natal	29˚31'36.3"S 30˚15'27.4"E
			Howick, KwaZulu-Natal	29˚31'39.9"S 30˚15'13.3"E
			Boston Park, KwaZulu-Natal	29˚41'24.4"S 30˚03'53.3"E
Ceratitis pedestris	*Strychnos cocculoides*	Loganiaceae	Bela Bela, Limpopo	24˚53'17"S 28˚23'16"E
	Strychnos pungens	Loganiaceae	Bela Bela, Limpopo	24˚51'55"S 28˚23'31"E
Ceratitis podocarpi	*Afrocarpus falcatus*	Podocarpaceae	Groenkloof, Pretoria, Gauteng	25˚46'20.2"S 28˚13'17.8"E
			University of Pretoria, Pretoria, Gauteng	25˚45'12.2"S 28˚13'38.4"E
			Brooklyn, Pretoria, Gauteng	25˚46'36.5"S 28˚13'39.3"E
Ceratitis scaevolae	*Scaevola plumieri*	Goodeniaceae	Richards Bay, KwaZulu-Natal	29˚27'21.0"S 31˚16'57.6"E
			Tinley Manor, KwaZulu-Natal	29˚27'21"S 31˚16'57"E

Table S3. Number of hosts confirmed for each of the eight species, and group size for females and males assayed in the survival experiment. Host records were obtained from De Meyer *et al.* (2002) and an online identification key (fruitflykeys.africamuseum.be). Sample size does not include individuals used for biochemical assays. The censored flies are the individuals that escaped or did not die from natural death (e.g. stuck in the sugar that tends to become liquid with time). They were not removed from the data set but were instead counted as censored in the survival analysis.

Species	Number of host (families)	Sample size (F / M)	Flies censored (F / M)
Bactrocera dorsalis	305 (59)	37 / 43	4 / 1
Ceratitis capitata	194 (53)	50 / 35	-
Ceratitis quilicii	13 (5)	86 / 90	- / 4
Ceratitis cosyra	10 (7)	47 / 73	1 / 1
Ceratitis rubivora	9 (1)	42 / 36	-
Ceratitis pedestris	6 (1)	54 / 71	3 / 9
Ceratitis podocarpi	3 (1)	33 / 36	-
Ceratitis scaevolae	1 (1)	85 / 73	4 / 3

Table S4. Origin and identification number for each individual of each species used to build the phylogenetic tree.

Species	ID	Country of origin	Database
Bactrocera dorsalis	AGIMP023-13	India	Bold Systems
	GBDPT1331-15	Nigeria	Bold Systems
	GBMIN62907-17	Nigeria	Bold Systems
	GBMIN62909-17	Nigeria	Bold Systems
	GBMIN62911-17	Nigeria	Bold Systems
	GBMIN62912-17	Nigeria	Bold Systems
	GBMIN62914-17	Nigeria	Bold Systems
Ceratitis capitata	GBDP6963-09	South Africa	GenBank
	GBDP6960-09	South Africa	GenBank
	GBDP6956-09	South Africa	GenBank
	GBDP6887-09	South Africa	GenBank
	GBDP6886-09	South Africa	GenBank
	GBDP1758-06	Kenya	GenBank

	GBDP17543-15	Kenya	GenBank
Ceratitis quilicii	KU688181	Tanzania	GenBank
	KU688187	South Africa	GenBank
	KU688192	Reunion Island	GenBank
	KU688197	Tanzania	GenBank
	KU688201	South Africa	GenBank
Ceratitis cosyra	GBDP2218-06	South Africa	GenBank
	GBDP2219-06	South Africa	GenBank
	GBDP10791-12	Mozambique	GenBank
	GBMIN32872-13	South Africa	GenBank
	JTB020-12	Mozambique	Bold Systems
	JTB277-12	Botswana	Bold Systems
	MVTBI225-08	Mozambique	Bold Systems
	MVTBI226-08	Mozambique	Bold Systems
	MVTBI227-08	Mozambique	Bold Systems
Ceratitis rubivora	GBDP10816-12	South Africa	GenBank
	GBMIN19472-13	Kenya	GenBank
	GBMIN19473-13	Kenya	GenBank
	GBMIN19474-13	Kenya	GenBank
	GBMIN19592-13	Kenya	GenBank

	GBMIN19593-13	Kenya	GenBank
	GBMIN32848-13	South Africa	GenBank
	JTB234-12	Tanzania	Bold Systems
	MVTBI078-08	South Africa	Bold Systems
Ceratitis pedestris	GBDP10805-12	Kenya	GenBank
	GBDP17558-15	Kenya	GenBank
	GBMIN32860-13	Benin	GenBank
	JTB021-12	Mozambique	Bold Systems
	JTB326-12	Mozambique	Bold Systems
	MVTBI061-08	Mozambique	Bold Systems
	MVTBI318-09	Mozambique	Bold Systems
Ceratitis podocarpi	GBDP10806-12	South Africa	GenBank
	GBMIN19465-13	Kenya	GenBank
	GBMIN19466-13	Kenya	GenBank
	GBMIN19584-13	Kenya	GenBank
	GBMIN19585-13	Kenya	GenBank
	GBMIN32859-13	Kenya	GenBank

	MVTBI062-08	Kenya	Bold Systems
	MVTBI063-08	Kenya	Bold Systems
Ceratitis scaevolae	CSCA001-18	South Africa	Bold Systems
	CSCA002-18	South Africa	Bold Systems
	CSCA003-18	South Africa	Bold Systems

Table S5. Fruit phenology of the South African host plants associated with the eight fruit flies species.

	Family	Latin name	Ref	Jan	Feb	Mar	Apr	May	Jun	Jul	Aug	Sep	Oct	Nov	Dec
C. capitata	Anacardiaceae	*Harpephyllum caffrum*	1	■	■	■	■	■	■	■	■	■			■
	Anacardiaceae	*Mangifera indica*	2	■	■	■									
	Apocynaceae	*Carissa bispinosa*	1	■	■	■	■	■	■	■	■	■	■		
	Apocynaceae	*Carissa edulis*	1	■										■	■
	Apocynaceae	*Carissa grandiflora*	2	■	■	■	■	■	■	■	■	■	■	■	■
	Cactaceae	*Pereskia aculeata*	2	■	■	■	■	■	■	■	■	■	■	■	■
	Capparaceae	*Capparis sepiaria*	1	■	■	■	■	■	■						
	Chrysobalanaceae	*Parinari curatellifolia*	1	■									■	■	■

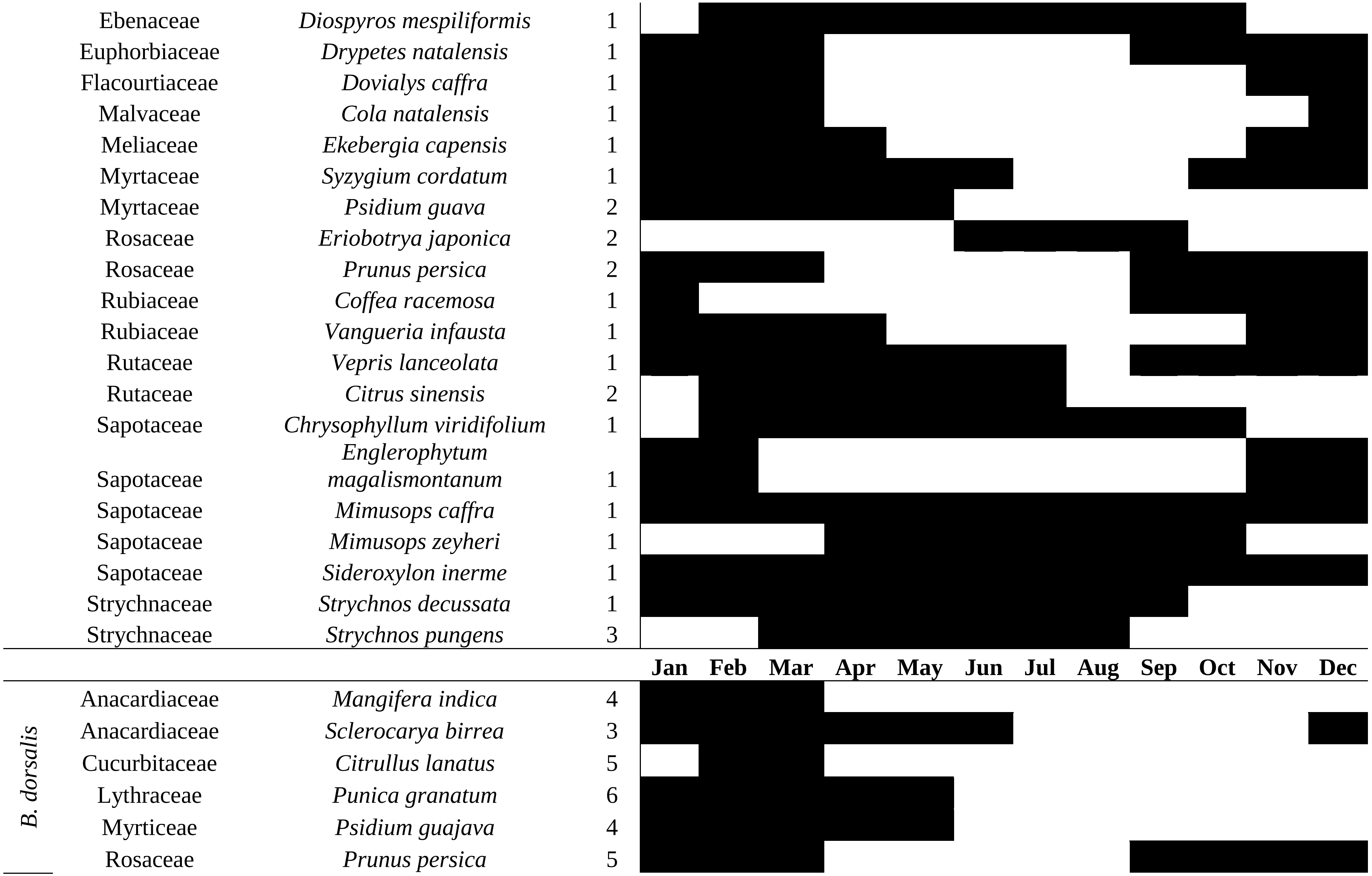
Ebenaceae | Diospyros mespiliformis | 1
Euphorbiaceae | Drypetes natalensis | 1
Flacourtiaceae | Dovialys caffra | 1
Malvaceae | Cola natalensis | 1
Meliaceae | Ekebergia capensis | 1
Myrtaceae | Syzygium cordatum | 1
Myrtaceae | Psidium guava | 2
Rosaceae | Eriobotrya japonica | 2
Rosaceae | Prunus persica | 2
Rubiaceae | Coffea racemosa | 1
Rubiaceae | Vangueria infausta | 1
Rutaceae | Vepris lanceolata | 1
Rutaceae | Citrus sinensis | 2
Sapotaceae | Chrysophyllum viridifolium | 1
Sapotaceae | Englerophytum magalismontanum | 1
Sapotaceae | Mimusops caffra | 1
Sapotaceae | Mimusops zeyheri | 1
Sapotaceae | Sideroxylon inerme | 1
Strychnaceae | Strychnos decussata | 1
Strychnaceae | Strychnos pungens | 3
Jan Feb Mar Apr May Jun Jul Aug Sep Oct Nov Dec
B. dorsalis
Anacardiaceae | Mangifera indica | 4
Anacardiaceae | Sclerocarya birrea | 3
Cucurbitaceae | Citrullus lanatus | 5
Lythraceae | Punica granatum | 6
Myrticeae | Psidium guajava | 4
Rosaceae | Prunus persica | 5

	Family	Species		Jan	Feb	Mar	Apr	May	Jun	Jul	Aug	Sep	Oct	Nov	Dec
	Rosaceae	*Eriobotrya japonica*	5						■	■	■	■			
	Rubiaceae	*Vangueria infausta*	4	■	■	■	■							■	■
	Rutaceae	*Citrus sinensis*	4		■	■	■	■	■	■					
	Salinaceae	*Flacourtia indica*	5	■	■	■	■	■	■						
	Solanaceae	*Solanum mauritanum*	4	■	■	■	■	■	■	■	■	■	■	■	■
				**Jan**	**Feb**	**Mar**	**Apr**	**May**	**Jun**	**Jul**	**Aug**	**Sep**	**Oct**	**Nov**	**Dec**
C. quilicii	Anacardiaceae	*Harpephyllum caffrum*	2	■	■	■	■	■	■	■	■	■			■
	Apocynaceae	*Carissa grandiflora*	2	■	■	■	■	■	■	■	■	■	■	■	■
	Chrysobalanaceae	*Parinari curatellifolia*	1	■									■	■	■
	Flacourtiaceae	*Dovialys caffra*	1	■	■	■								■	■
	Flacourtiaceae	*Rawsonia lucida*	2	■	■									■	■
	Malvaceae	*Cola natalensis*	1	■	■	■									■
	Meliaceae	*Ekebergia capensis*	2	■	■	■	■								■
	Moraceae	*Ficus carica*	2	■	■								■	■	■
	Myrtaceae	*Psidium guava*	2	■	■	■	■	■							
	Myrtaceae	*Syzygium cordatum*	1	■	■	■	■	■	■				■	■	■
	Rosaceae	*Prunus persica*	2	■	■	■						■	■	■	■
	Sapotaceae	*Englerophytum magalismontanum*	1	■	■									■	■
	Sapotaceae	*Englerophytum natalense*	1						■	■	■	■	■	■	■
	Solanaceae	*Solanum giganteum*	3	■	■	■	■	■	■	■					■
	Strychnaceae	*Strychnos spinosa*	3			■	■	■	■	■	■	■	■	■	
				**Jan**	**Feb**	**Mar**	**Apr**	**May**	**Jun**	**Jul**	**Aug**	**Sep**	**Oct**	**Nov**	**Dec**
C. cosyra	Anacardiaceae	*Harpephyllum caffrum*	7	■	■	■	■	■	■	■	■	■			■
	Anacardiaceae	*Mangifera indica*	2	■	■	■									
	Anacardiaceae	*Sclerocarya birrea*	3	■	■	■	■	■	■						■
	Annonaceae	*Annona senegalensis*	2	■	■	■						■	■	■	■
	Canellaceae	*Warburgia salutaris*	1	■									■	■	■

				Jan	Feb	Mar	Apr	May	Jun	Jul	Aug	Sep	Oct	Nov	Dec
	Chrysobalanaceae	*Parinari curatellifolia*	1	■									■	■	■
	Fabaceae	*Cordila africana*	1	■	■	■								■	■
	Flacourtiaceae	*Dovialys caffra*	1	■	■	■								■	■
				**Jan**	**Feb**	**Mar**	**Apr**	**May**	**Jun**	**Jul**	**Aug**	**Sep**	**Oct**	**Nov**	**Dec**
C. pedestris	Strychnaceae	*Strychnos gerrardi*	2		■	■	■	■	■	■	■	■	■	■	
	Strychnaceae	*Strychnos pungens*	2			■	■	■	■	■	■				
	Strychnaceae	*Strychnos spinosa*	2			■	■	■	■	■	■	■	■	■	
	Strychnaceae	*Strychnos cocculoides*	7				■	■	■	■	■				
				**Jan**	**Feb**	**Mar**	**Apr**	**May**	**Jun**	**Jul**	**Aug**	**Sep**	**Oct**	**Nov**	**Dec**
C. rubivora	Rosaceae	*Rubus fruticosus*	2	■	■									■	■
				**Jan**	**Feb**	**Mar**	**Apr**	**May**	**Jun**	**Jul**	**Aug**	**Sep**	**Oct**	**Nov**	**Dec**
C. podocarpi	Podocarpaceae	*Afrocarpus falcatus*	7	■	■	■	■	■	■	■	■	■	■	■	■
				**Jan**	**Feb**	**Mar**	**Apr**	**May**	**Jun**	**Jul**	**Aug**	**Sep**	**Oct**	**Nov**	**Dec**
C. scaevolae	Goodenaceae	*Scaevola plumieri*	2	■	■									■	■

The table only displays hosts that are associated with the given fruit fly species in South Africa and for which information on the fruit phenology could be found. The reference numbers indicate the source mentioning the association between the host and the fruit fly species. 1) Grové *et al.* (2016); 2) De Meyer *et al.* (2002); 3) Grové *et al.* (2017); 4) Theron *et al.* (2017); 5) Mwatawala *et al.* (2006); 6) Jose *et al.* (2013); 7) Personal observation.

References:

De Meyer, M., Copeland, R.S., Lux, S.A., Mansell, M., Quilici, S., Wharton, R., White, I.M. & Zenz, N.J. 2002. Annotated check list of host plants for afrotropical fruit flies (Diptera: Tephritidae) of the genus *Ceratitis*. *Documentation Zoologique du Musee Royale de l'Afrique Centrale* **27:** 1-91.

Grové, T., De Jager, K., De Beer, M.S. & Hannweg, K. 2016. Indigenous host plants for economic importance fruit flies species in South Africa. In: *South African grower's association yearbook*, Vol. 39, pp. 19-23.

Grové, T., De Jager, K. & De Beer, M.S. 2017. Indigenous hosts of economically important fruit fly species (Diptera: Tephritidae) in South Africa. *J Appl Entomol* **141:** 817-824.

Jose, L., Cugala, D. & Santos, L. 2013. Assessment of Invasive fruit fly infestation and damage in Cabo Delgado Province, Northern Mozambique. *African Crop Science Journal* **21:** 21-28.

Mwatawala, M.W., De Meyer, M., Makundi, R.H. & Maerere, A.P. 2006. Seasonality and host utilization of the invasive fruit fly, Bactrocera invadens (Dipt., Tephritidae) in central Tanzania. *J Appl Entomol* **130:** 530-537.

Theron, C.D., Manrakhan, A. & Weldon, C.W. 2017. Host use of the oriental fruit fly, Bactrocera dorsalis (Hendel) (Diptera: Tephritidae), in South Africa. *J Appl Entomol* **141:** 810-816.
